# Perish or Publish?

**DOI:** 10.1055/a-2283-2269

**Published:** 2024-05-09

**Authors:** Joon Pio Hong, Geoffrey G. Hallock

**Affiliations:** 1 Department of Plastic and Reconstructive Surgery, Asan Medical Center, University of Ulsan College of Medicine, Seoul, Republic of Korea; 2 Division of Plastic Surgery, Sacred Heart Campus, St. Luke's Hospital, Allentown, Pennsylvania

**Figure:**
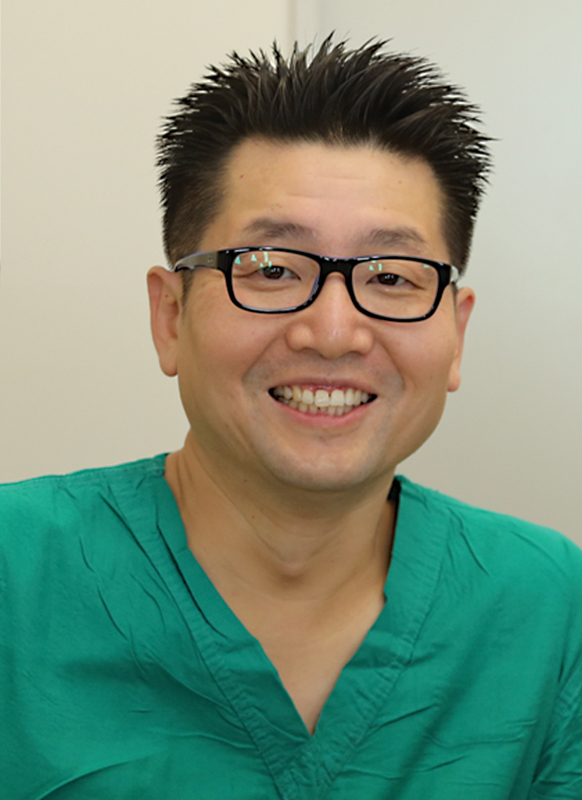
JP Hong: Editor-in-Chief

**Figure:**
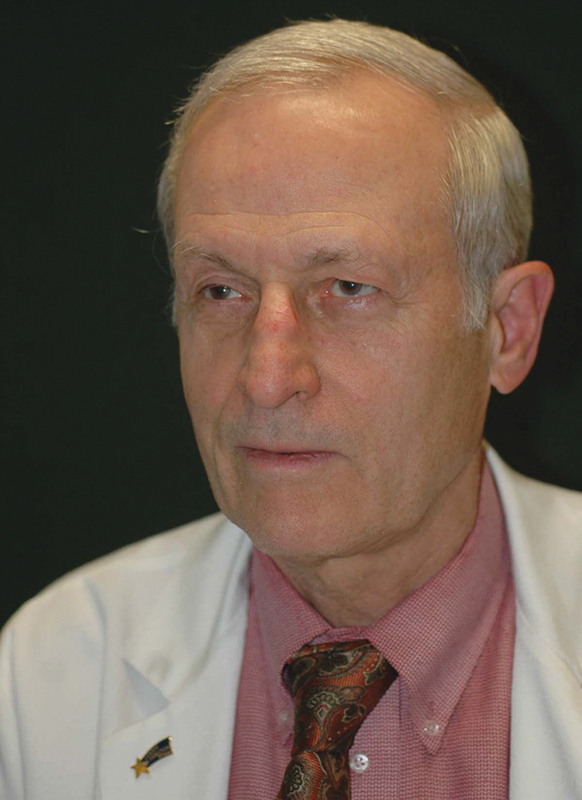
Geoffrey G. Hallock: Associate Editor

“The best surgical instrument is the pencil with which the surgeon records his [sic. her] thoughts…”

–Dieffenbach 1

Do we just have dyslexia or perhaps are we foretelling? The converse idiom *publish or perish* is not just entrenched in our own profession of medicine, but pervasive throughout academia whether found in the other natural sciences or even the social sciences. 2 3 4 5 6 This phrase was not born yesterday, but has been stated to have originated with the sociologist Wilson as long ago as 1942. 4 Other variations with the same theme are ubiquitous—“ *publish and perish* ,” 7 8 “ *publish and still perish* ,” 9 “ *to publish or not to publish* ,” 10 11 “ *publish or be ethical* ,” 12 “ *why publish?* ” 13 14 “ *publish for purpose* ,” 15 as a short list. We both profess to be scholarly and try to be teachers, so we have always enjoyed the pleasure of transforming our thoughts and ideas into mere words. 14 Just as a carpenter builds things from wood and stone, our purpose has been to use the medium of publishing to achieve our objective, whether it be at conferences or meetings, via books or book chapters, or just journal articles as you might suppose. Writing is only one aspect of communication, where guided also by speech and symbols—and plastic surgeons at the least enjoy their pictures—this must be based on skills as Tempest said in his Gillies lecture (1987) that must be learned and mastered, predicated in science or our teaching will be incorrect, and the training we give our students or even ourselves will be without imagination or innovation. 16

There are moral reasons to publish. The requisite research for whatever format to be used will ensure that we function at a current “state of the art” level recognizing the latest findings in our endeavor. 7 Any advancement of knowledge gained should be disseminated so that that can be utilized as applicable throughout the world today, 7 realizing we are just a small part thereof. The ultimate goal of evidence-based medicine and outcome studies must always be the improvement of patient care. 5 15 17 18 Dare we say that replication studies, albeit at greater risk for being denied publishing, are still essential to criticize or question, or just as important to corroborate the prior findings of our colleagues. 14 15 Of course, we as other authors in this process will always witness our own personal intellectual as well as professional development, 14 while at the same time always having “fun” whether writing with domestic or international colleagues or mentoring our own fellows. 11 17 Remember, we both have incessantly, repeatedly, and boringly stated while in the role of teacher that whatever your innovation, “if you haven't written it, you haven't done it.” So do it.

Unfortunately, we do realize there are other reasons to publish. Long ago, educational or research quality, public service, or priority in discovery were alone the incentives. 4 Today, these have been displaced by more ominous rewards. Medical students seeking highly competitive residencies (some say even in plastic surgery) have early on recognized that first authorship in high-impact journals will be an asset in the application process. 17 19 20 Hiring policies whether for graduate programs or into the university hierarchy, career advancement, academic promotion and tenure, and even obtaining research grants may be judged on peer-reviewed output alone, 2 7 11 15 17 18 19 21 or impact factors or citation metrics that may find their way into the curriculum vitae, 4 7 19 21 22 as quantity is easier to measure than quality. 14 To compound the problem, department, hospital, university, or even government status and reputation may depend on just numbers alone. 4 7 11 15 19

The “dark side” of all this we all must endure in addition to that inherent with the practice of surgery are the additional personal stress, feelings of insecurity as we are just “providers,” dealing with our own health issues, and for some substance abuse. 14 These pressures to publish prolifically have disregarded the tsunami of trivialities or conformity in so-called “safe” research projects that are less likely to be rejected 15 that have swept past us. This escapism has walked hand-in-hand with unethical publication practices that would include plagiarism, fabrication, falsification of data, or the exponential growth of “ghost” authorships. 8 15 23 24 Artificial intelligence, or for some alternative intelligence, has opened the door wider for continuing mass production of these same problems, as originality and creativity will be lost since their content by definition must be based on data that already exist and not innovation. 5 15 Granted, AI will be a boon for the non-native English-speaking researcher and others where translation capabilities, grammar checks, or compilation of sometimes appropriate references will be more efficiently possible. 5

Online access (OA) must be further overviewed, as has this journal serviced admirably so many readers, yet the concept has resulted in a multitude of “predatory” 3 17 or “vanity” 25 journals that aim not at academic rigor nor undergo peer review; but rather solely exist to generate income. As such, since publication may be rapid without need for revision, this may provide the author the services they desire to accelerate promotion or tenure, understandably why the nomenclature for some is to be “zombie professors.” 3 This course is all undertaken at the risk that lower such journal standards and reputation will diminish the possibility for citation if the article can even be found to be read. 3 Caution is advised, as legitimate OA journals are usually affiliated with an established scholarly society or academic institution, and have a dedicated editorial staff among other attributes. 3 Remember when embarking on this journey to publish what Burbules says, 14 the writer in general must accept four rules with regards to the reader:

1. Half of all manuscripts published are read by no more than the authors, reviewers, and editors.
2. Most who do read, will not understand you the way you want.
3. Most who do read, and do understand, will disagree with what you have said.
4. Most who disagree, will be for reasons you find disagreeable.

Other beneficial internet sources, in some way, that cannot be overlooked include the software program constantly upgraded by Harzing 6 entitled appropriately “ *Publish or Perish.* ” Using this, the individual can have help in their literature review, calculate citation metrics optimally even if citations are few, decide which journal would be preferable for manuscript submission, or even in preparation for a job interview. 6 Not to be disregarded also must be social media. Social media outlets can communicate your ideas far quicker and to a far greater number of surgeons than the average journal article that probably is never read. 14 Debate and discourse herein that will advance the desired learning and development can be encouraged. 26 Just ask this junior author as to the validity of this variation of “publishing.” As Lindsay et al 26 predict, the soon archaic phrase “ *publish or perish* ,” a synonym for personal achievement, will evolve to “ *have presence or perish* .” Anonymity will intentionally be forsaken, so there will be greater certainty that your work more likely will be read or heard. 26 And who was Gillies? 16
